# Heterogeneity in Neutrophil Extracellular Traps from Healthy Human Subjects

**DOI:** 10.3390/ijms25010525

**Published:** 2023-12-30

**Authors:** Margaret S. Collins, Michelle A. Imbrogno, Elizabeth J. Kopras, James A. Howard, Nanhua Zhang, Elizabeth L. Kramer, Kristin M. Hudock

**Affiliations:** 1 Division of Pulmonary, Critical Care & Sleep Medicine, Department of Medicine, University of Cincinnati College of Medicine, Cincinnati, OH 45267, USA; 2 Department of Pharmacology & Systems Physiology, University of Cincinnati, Cincinnati, OH 45267, USA; 3 Department of Pediatrics, University of Cincinnati College of Medicine, Cincinnati, OH 45229, USA; 4 Division of Biostatistics and Epidemiology, Cincinnati Children’s Hospital Medical Center, Cincinnati, OH 45229, USA; 5 Division of Pediatric Pulmonary Medicine, Cincinnati Children’s Hospital Medical Center, Cincinnati, OH 45229, USA; 6 Division of Pulmonary Biology, Cincinnati Children’s Hospital Medical Center, Cincinnati, OH 45229, USA

**Keywords:** NET heterogeneity, neutrophil extracellular traps, DNA, neutrophil elastase

## Abstract

Neutrophil extracellular traps (NETs), a key component of early defense against microbial infection, are also associated with tissue injury. NET composition has been reported to vary with some disease states, but the composition and variability of NETs across many healthy subjects provide a critical comparison that has not been well investigated. We evaluated NETs from twelve healthy subjects of varying ages isolated from multiple blood draws over a three-and-one-half-year period to delineate the variability in extracellular DNA, protein, enzymatic activities, and susceptibility to protease inhibitors. We calculated correlations for NET constituents and loss of human bronchial epithelial barrier integrity, measured by transepithelial electrical resistance, after NET exposure. We found that although there was some variability within the same subject over time, the mean NET total DNA, dsDNA, protein, LDH, neutrophil elastase (NE), and proteinase 3 (PR3) in isolated NETs were consistent across subjects. NET serine protease activity varied considerably within the same donor from day to day. The mean NET cathepsin G and MPO were significantly different across donors. IL-8 > IL-1RA > G-CSF were the most abundant cytokines in NETs. There was no significant difference in the mean concentration or variability of IL-8, IL-1RA, G-CSF, IL-1α, IL-1β, or TNF-α in different subjects’ NETs. NET DNA concentration was correlated with increased NET neutrophil elastase activity and higher NET IL-1RA concentrations. The mean reduction in protease activity by protease inhibitors was significantly different across donors. NET DNA concentration correlated best with reductions in the barrier integrity of human bronchial epithelia. Defining NET concentration by DNA content correlates with other NET components and reductions in NET-driven epithelial barrier dysfunction, suggesting DNA is a reasonable surrogate measurement for these complex structures in healthy subjects.

## 1. Introduction

Neutrophils provide a first line of defense against infection [1]. Polymorphonuclear leukocytes (PMNs) are actively recruited to the site of injury or infection by chemoattractants produced by the host and by invading microbes [2,3]. Stimulated PMN responses include phagocytosis, release of oxidative burst, and expulsion of neutrophil extracellular traps (NETs) [4]. NET formation occurs when histone arginine and methylarginine are converted to citrulline, leading to structural changes in the neutrophil chromatin [5,6]. Subsequently, the decondensation of nuclear contents and expansion of the nuclei lead to an expulsion of NETs via NETosis [7]. 

NETs are increased in inflammatory lung diseases, including cystic fibrosis (CF) and COPD, and are associated with the severity of lung disease; however, little is known regarding how NETs drive disease pathogenesis [8,9,10]. NETs are comprised of DNA strands studded with bioactive compounds such as proteases [5]. Serine proteases neutrophil elastase (NE), proteinase 3 (PR3), and cathepsin G (CG) are present in NETs and can be bactericidal [11]. Excess NE is associated with worse structural lung disease in CF and COPD [12]. We have demonstrated that NET serine proteases cause significant bystander tissue damage, as exposing normal human bronchial epithelial cells (HBE) to NETs led to a breakdown in epithelial barrier integrity, including decreases in transepithelial electrical resistance (TEER) [13]. We have shown that NET exposure also drives inflammation through NE by activating the IL-1 pathway in the epithelium, leading to the secretion of TNF-α and IL-8 [14]. Protease inhibitors present a crucial therapeutic option to blunt protease-related tissue destruction; however, the susceptibility of individual subjects’ NETs to chemical or naturally occurring antiprotease molecules has not been established. The cytokine IL-8 is another immunomodulatory protein present in NETs and is a potent chemoattractant for neutrophils [14,15]. While the relative abundance of the protein components has been reported, little is known about the variability in NET composition and enzymatic activities across healthy human NET donors [16]. Likewise, the degree to which these components correlate with NET-induced lung tissue injury has not been investigated. 

In this study, we sought to characterize isolated NETs from normal human donors, including analysis of DNA and protein content, enzymatic activities, and susceptibility to selective inhibitors. We then correlated these NET components with the loss of barrier integrity in NET-exposed HBE. The rationale for conducting this study was to fill a gap in the current literature regarding the variability in NET composition and enzymatic activities across healthy human NET donors and to determine the extent to which specific NET components correlate with loss of resistance and disruption of the lung epithelial monolayer. Determining the NET content is essential because the composition likely drives the biological impact of the NET, and studying healthy subjects’ NETs provides a critical foundation to establish the normal spectrum for comparison of NETs from diseased patients in future studies. We hypothesized that the DNA content of NETs would correlate with the concentration of other key NET components and could serve as a surrogate measurement for the complex NET structure.

## 2. Results

### 2.1. Mean Neutrophil Numbers Are Consistent across Subjects

**Figure 1:**
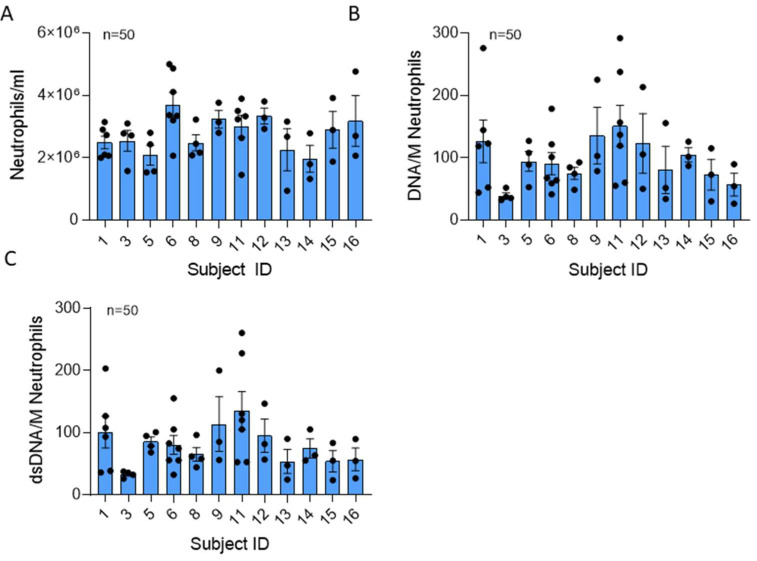
Neutrophil number and total extracellular DNA content of NETs are similar across donors. (**A**) Mean number of neutrophils recovered per ml blood drawn from 12 healthy donors in 50 collections using negative bead selection was not significantly different between donors (*p* = 0.068). (**B**) Mean NET DNA and (**C**) dsDNA concentrations expressed as ng DNA/million neutrophils was not significantly different between donors (*p* = 0.125 and 0.540, respectively). Data analyzed across subjects by Kruskal Wallis.

We freshly collected peripheral blood from 12 healthy male and female subjects over 3 1/2 years from 2020 to 2023. Donor characteristics and the timing of blood collections for each donor are described in Table 1 and Supplemental Table S1, respectively. Our population included 50% men with a mean age of 41.2 ± 17.4 years old and 50% women with a mean age of 37.8 ± 16.5 years old. To determine which NET components are likely to cause lung tissue injury, we sought to delineate the extent to which NET content varies across different healthy donors and within the same donor on different days. Isolated human NETs were assessed for DNA content, total protein concentration, lactate dehydrogenase (LDH), serine proteases (NE, CG, and PR3), myeloperoxidase (MPO), and cytokines. In Figure 1A, neutrophils per milliliter of peripheral blood from healthy donors were compared. Neutrophil concentrations ranged from 9.4 × 10^5^ to 5 × 10^6^/mL, and the mean number of neutrophils isolated was consistent across donors. There was no significant difference in variability across donors (*p* = 0.89).

### 2.2. NET DNA Content Is Similar across Donors

DNA serves as the backbone of NETs. Total extracellular DNA and double-stranded DNA (dsDNA) measurements of each NET preparation were averaged and expressed as ng DNA per 10^6^ neutrophils (Figure 1B). Average DNA content ranged from 25.9 to 291.9 ng/million neutrophils, with a mean NET DNA of 109.73 ± 182.17 ng/mL. The mean NET DNA content was not significantly different across subjects (*p* = 0.54). Four of seven donors exhibited variability in DNA from day to day, suggesting that some subjects’ neutrophils may vary in their capacity to form NETs. 

Interestingly, dsDNA represents an average of 29.6 ± 30.32% of the total DNA found in NETs generated from healthy humans (Figure 1B,C). The dsDNA content range was 19.2 ± 260.4 ng/million neutrophils, and the mean dsDNA was not significantly different across donors. Variability in dsDNA content was not significant between subjects (*p* = 0.33). 

### 2.3. NET Protease Activity Is Consistent across Healthy Subjects

**Figure 2:**
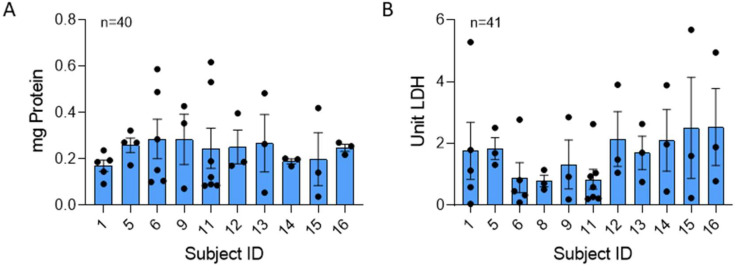
Total protein and LDH is consistent across human NETs. Mean values of protein and LDH were compared across 40 and 41 blood draws from 10 and 11 donors, respectively. Samples were normalized to µg NET DNA. (**A**) No significant difference was found in protein concentration between donors (*p* = 0.933). (**B**) LDH levels, also normalized to NET DNA, were not significantly different across donors (*p* = 0.584). Data analyzed across subjects by Kruskal Wallis.

The NET structure carries a payload of antimicrobial proteins. The total protein content of isolated NETs was measured and normalized to µg DNA of each sample (Figure 2A). The mean total protein in the NETs was not significantly different between donors. There was no significant variability across the group (*p* = 0.51), but there was variability within the same subject from day to day for a few donors (06, 09, 11). 

LDH was consistently present in our isolated NETs produced by stimulation of neutrophils with 25 nM PMA. LDH is released into the supernatant in response to cell membrane damage and is an indicator of cytotoxicity [17]. There was no significant difference in the mean or variability (*p* = 0.81) in NET LDH content across individuals (Figure 2B).

**Figure 3:**
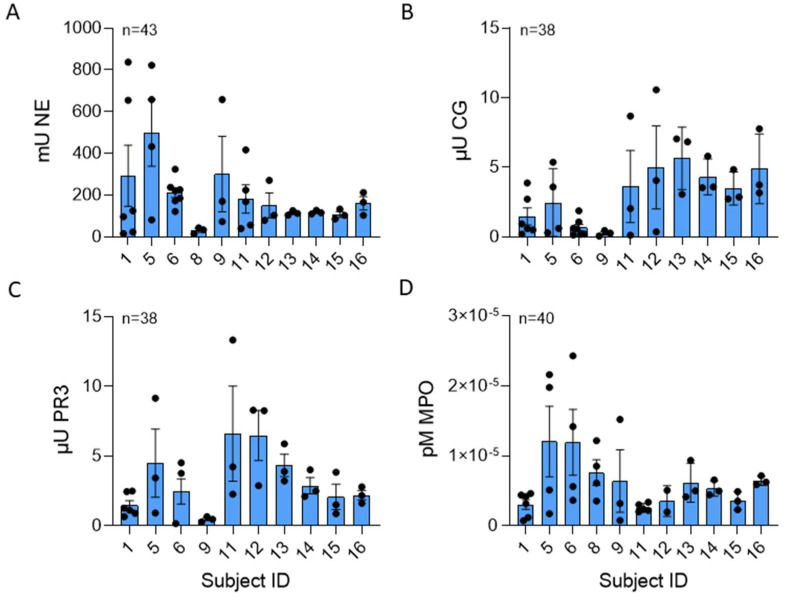
CG and MPO NET enzyme activity varied across healthy subjects. (**A**) Human NE (*p* = 0.194), (**B**) CG (*p* = 0.034), (**C**) PR3 (*p* = 0.057), and (**D**) MPO (*p* = 0.011) enzyme activities were measured in NETs isolated from up to 11 normal donors between 38 to 43 blood draws and normalized per µg DNA. NE, CG, and MPO data analyzed across subjects by Kruskal Wallis, PR3 data analyzed by one-way ANOVA.

Serine proteases NE, CG, and PR3, stored in the azurophilic granules of PMNs, are structurally related, have antibacterial properties, and are critical to the destruction of pathogens [1]. These bioactive compounds are part of the milieu of proteins associated with NET DNA. In Figure 3A–D, the greatest serine protease activity was contributed by NE, with a mean of 35.04 ± 81.6 mU/µg NET DNA. CG and PR3 concentrations were 1.56 ± 6.99 µU/µg and 4.4 ± 20.5 µU NET DNA, respectively. The mean NE and PR3 activities were not different; however, the mean CG activity was significantly different across donors. Although there was no statistically significant difference in variability across donors for NE (*p* = 0.16), CG (*p* = 0.32), or PR3 (0.717) activities, there was variability within repeated measures from the same NET donor (NETs isolated on different days). MPO is also stored in azurophilic granules, is antimicrobial, can be a source of inflammation, and can be involved in NET formation [18,19]. The mean MPO activity measured 7.99 ± 1.63 × 10^−5^ pM/min/mL per μg NET DNA, and there was a significant difference in MPO activity across donors. There was no significant difference in MPO variability across donors (*p* = 0.52). Age and gender were not found to be significantly associated with any of the NET components (all *p*-values > 0.05). 

**Figure 4:**
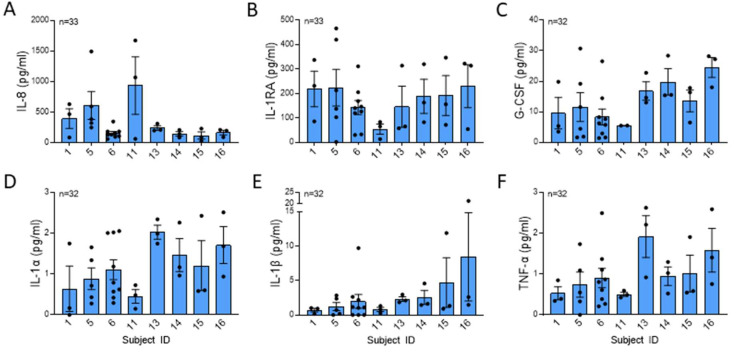
Cytokines were not significantly different across healthy subjects. (**A**) IL-8 (*p* = 0.096), (**B**) IL-1RA (*p* = 0.646), (**C**) G-CSF (*p* = 0.132), (**D**) IL-1α (*p* = 0.249), (**E**) IL-1β (*p* = 0.200), and (**F**) TNF-α (*p* = 0.314) were measured in isolated NETs by Luminex from 8 subjects from 32 or more blood draws and were normalized per µg DNA. IL-1RA data analyzed across subjects by one-way ANOVA and all other cytokine data analyzed by Kruskal Wallis.

NETs contribute cytokines to the extracellular milieu. Ref. [20] found that NET-bound cytokines could contribute to a positive feedback loop in inflammation. The most abundant cytokines in isolated NETs were IL-8, a potent neutrophil chemoattractant, IL-1RA, a competitive antagonist for the IL-1 receptor 1, and G-CSF, a potent stimulator for the bone marrow to increase neutrophil production (Figure 4A–C) [3,15,21]. Proinflammatory cytokines IL-1α, IL-β, and TNFα were present in NETs (Figure 4D–F). Although there was a trend towards a difference in the mean concentration of NET IL-8, there were no statistically significant differences in mean cytokine concentrations or variability across the subjects.

**Figure 5:**
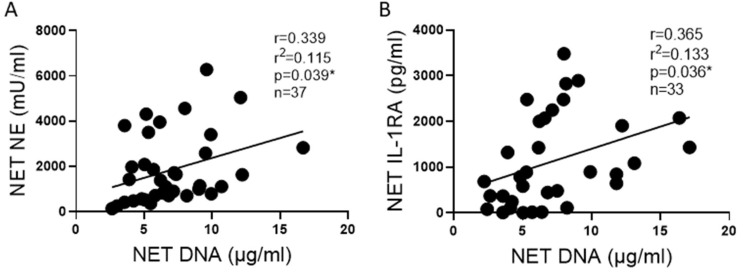
NE and IL-1RA correlate with NET DNA concentration. (**A**) NE activity correlates with DNA concentrations in NETs from 11 blood donors across 37 blood draws. (**B**) The anti-inflammatory cytokine IL-1RA concentration correlates with DNA concentrations in NETs from 11 blood donors across 33 blood draws. Data analyzed by Pearson correlation with best fit line by simple linear regression * *p* < 0.05.

Next, we sought to determine if enzyme activities or cytokine concentrations correlated with the DNA NET concentration. We found that NET NE activity positively correlated with NET DNA concentrations (Figure 5A). Likewise, a positive correlation was observed between IL-1RA and DNA concentrations (Figure 5B).

### 2.4. Susceptibility to Serine Protease Inhibitors Differs across Donors

**Figure 6:**
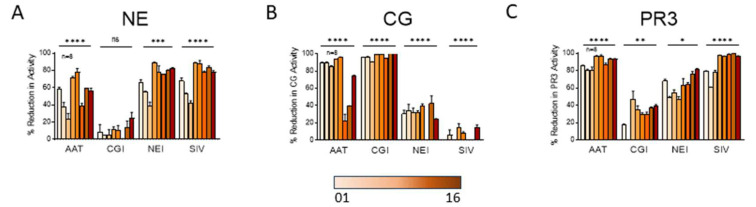
NETs from different subjects vary significantly in their susceptibility to protease inhibitors. In cell-free assays, 100 µg/mL protease inhibitors AAT, CGI, NEI, and SIV were incubated 18 h with NETs isolated from 8 donors. Samples were then assayed for (**A**) NE, (**B**) CG, and (**C**) PR3 activities and are expressed as percent reduction in enzymatic activity. Data analyzed across subjects by one-way ANOVA * *p* < 0.05, ** *p* < 0.01, *** *p* < 0.001, **** *p* < 0.0001.

NETs isolated from the peripheral blood of healthy donors were assessed for susceptibility to protease inhibitors or serpins (Figure 6A–C). Alpha-1 antitrypsin (AAT) is a naturally occurring irreversible protease pseudosubstrate that binds and inactivates serine proteases. AAT is also known to inhibit apoptosis and bind and inactivate IL-8 [22,23]. The CG inhibitor (CGI) is a selective and reversible inhibitor of CG, reported to have minimal effect on related proteases [24]. The NE inhibitor (NEI) is an N-benzoylindazole derivative that selectively targets the binding domain of NE [25]. Sivelestat (SIV) is a selective reversible inhibitor of NE [26]. 

The mean reductions in NE, CG, and PR3 activities were different across donors for most inhibitors (Figure 6A–C). We observed that some reportedly “selective inhibitors” cross-reacted and inhibited other serine proteases. For instance, CGI, which was highly effective in blocking CG activity in all donor NETs, also reduced PR3 activity to a lesser degree (Figure 6B,C). Notably, although we anticipated that AAT would decrease all serine proteases substantially, AAT had the greatest impact on PR3 activity (Figure 6C), a lesser effect on CG (Figure 6B), and the smallest impact on NE (Figure 6A). 

### 2.5. NET DNA Correlates with Reductions in Barrier Function

**Figure 7:**
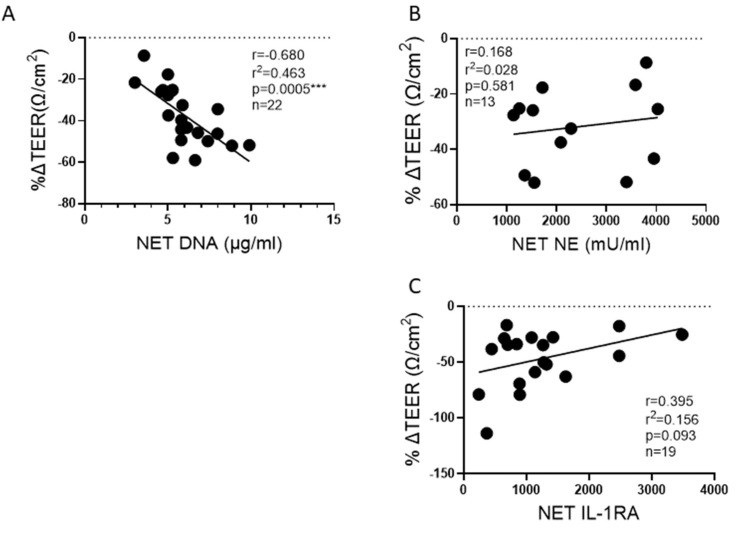
Correlation of NET components to change in lung epithelial barrier function. HBE grown at air-liquid interface (ALI) and exposed to PBS control or 5 μg/ml NETs from up to 9 donors across 13 to 22 blood draws. (**A**) The DNA concentration in NETs exposed to HBE significantly correlated to a reduction in TEER. (**B**) Activity of NE, the predominant serine protease in NETs, did not correlate with barrier function. (**C**) Increasing NET concentrations of the anti-inflammatory cytokine IL-1RA trended toward preservation of TEER. Data analyzed by Pearson correlation with best fit line by simple linear regression *** *p* < 0.001.

We have previously reported that HBE exposed to NETs have a significant decrease in barrier function, as measured by the TEER of epithelial monolayers [13]. However, it was unknown if NET components would consistently demonstrate similar associations across multiple donors. We, therefore, sought to determine which NET components from multiple donors correlated with reduced TEER. Increasing DNA concentrations in NETs negatively correlated with TEER in HBE (Figure 7A). NET NE activity did not correlate with reduced TEER (Figure 7B). IL-1RA concentrations in NETs trended towards correlating positively with TEER, which suggests IL-1RA in the NETs may offer a protective effect on the bronchial epithelia (Figure 7C). 

## 3. Discussion

Elevated NETs are present in many lung diseases including cystic fibrosis (CF), non-CF bronchiectasis, asthma, and COPD [8,9,10,27]. We and others have demonstrated that NETs can disrupt the barrier function of the epithelium and endothelium, a key mechanism of NET-induced pathogenesis in the lung [13,28]. NETs are complex structures with over 700 proteins, many of which are immunomodulatory, rendering defining NET concentrations challenging [29]. Herein, we describe the spectrum of NET contents for healthy human subjects and determine the critical components most associated with disrupted lung epithelial barrier function. 

NETs contain double- and single-stranded (ss) genomic DNA, which may have differential impacts on the host. We found that dsDNA was approximately two-thirds of the total DNA present in the NETs of healthy donors. The susceptibility of ssDNA or dsDNA to host endonucleases varies considerably, and ssDNA is often less stable. This may translate to NETs with greater dsDNA content having longer half-lives in vivo, amplifying their biological effect [30,31,32]. ssDNA and dsDNA can be perceived by the body differently and could elicit varying immune responses. “Uptake” of exogenous DNA by bacteria, e.g., Pseudomonas, can be beneficial to pathogens as DNA provides key nutrients for bacterial growth [33]. It is unclear if bacteria can more easily uptake ssDNA or dsDNA, but the NET ssDNA vs. dsDNA content could alter bacterial pathogenesis. 

LDH levels were high in the NETs of our human subjects. The amount of LDH we reported is very consistent across our healthy donors. The NET concentration of LDH may also be related to the stimulus for NETosis, for which we utilized PMA. Although PMA can cause necrosis of neutrophils, at the low doses used in this study, no necrosis was previously reported. LDH is widely used as a measure of cytotoxicity, but cytotoxicity may be overestimated in experiments if the NET contribution is not accounted for in the interpretation of the results [34].

There were differences in variability in NET enzymatic activity within the same person from day to day. The factors that regulate the extent of enzymatic activity within NETs on different days are unknown. NETs contain hundreds of components, and we previously demonstrated that exposure to single recombinant proteases NE, PR3, or CG did not recapitulate NET-induced reductions in HBE TEER [13]. These findings are consistent with the lack of correlation seen in Figure 7B between NET NE activity and changes in barrier function, a complex and dynamic biological process. We investigated susceptibility to protease inhibitors because we postulated that steric hindrance of the NET structure may limit direct binding and protease inactivation. We found significant differences in the effectiveness of protease inhibitors, which did not trend with the magnitude of protease activity. Interestingly, NE activity had the most variable response across donors to both irreversible (AAT) and reversible (SIV) inhibitors. NE has been shown to be the most abundant serine protease in NETs, and in this study, we demonstrated that NE is the greatest contributor to serine protease activity found in healthy subjects’ NETs. The reduction in NE activity by the inhibitors we tested was highly variable across subjects. The difference in variability across donors to NE inhibitors could have a considerable impact on the utility of anti-protease agents as therapies to reduce NET-induced pathology in CF or AAT deficiency. Further work will be needed to better understand what affects NET NE susceptibility to various protease inhibitors. 

We sought to describe the variability in NET composition across healthy donors to establish a foundation for future comparisons performed in diseased patients. We anticipate that NET composition will differ in disease states and could contribute to variable disease progression. One study of a single measurement of NET composition analyzed by mass spectrometry reported that subjects with lupus nephritis had increased methyl-oxidized α-enolase compared to controls [29]. In systemic lupus, ds-DNA is known to drive inflammation, and people with lupus often have elevated levels of autoantibodies to ds-DNA. It is possible that NETs derived from people with lupus have elevated ds-DNA content that triggers this pathology. 

Cytokine content in NETs was consistent across donors. IL-8 was the most abundant cytokine and is a critical neutrophil chemoattractant known to be elevated in many inflammatory lung diseases, including CF, alpha-1 antitrypsin deficiency, and acute respiratory distress syndrome (ARDS) [35,36,37,38]. In vivo, IL-8 can be free or bound, e.g., to immunoglobulin or heparin, which may affect the half-life and biologic impact [39,40]. It is unclear if IL-8 bound to DNA has similar caveats, and the extent to which a negatively charged DNA molecule could affect IL-8 interactions with the IL-8 receptor is unknown. Moreover, it is possible that the pool of IL-8 bound to DNA may not be detected to the same magnitude depending on the epitope by which IL-8 is detected for a given assay, thereby underestimating the IL-8 concentration of a given compartment. Another proinflammatory cytokine that we found in the NETs of our donors was G-CSF, which, to our knowledge, has not been previously reported. G-CSF is critical for neutrophil maturation and stimulating bone marrow to generate and release neutrophils. Both G-CSF and IL-8 can decrease neutrophil apoptosis, extending the neutrophil lifespan [41,42]. NETs also contained a considerable concentration of the anti-inflammatory cytokine IL-1RA. IL-1RA binds to the IL-1 receptor I but does not activate signal transduction as the IL-1α/β agonists do. We observed a trend towards a correlation between higher NET IL-1RA and reduced disruption of epithelial barrier function. It is possible that the IL-1RA in the NET may directly limit NET-induced barrier dysfunction by decreasing NET-driven IL-1α/β signaling. We previously demonstrated that IL-1α/β signaling drives epithelial secretion of TNF-α, which is known to alter epithelial tight junctions critical to barrier function [14,43].

A strength of our study is the inclusion of donors across the age spectrum and of both genders. Another strength was the study period of over 3 years, with multiple draws from donors. All donations were at least 1 month apart, hoping to minimize the impact of any single event on our results. We also made every effort to limit known factors that affect neutrophil function within our population, including collecting all blood donations within the same 2 h window in the morning. We recruited nonsmokers with no consumption of alcohol or NSAIDs within 3 days of donation and who had to have had greater than 2 weeks elapse after an acute illness or receiving antimicrobial therapy [44,45,46]. An equal number of similarly aged males and females were recruited. 

It would be impractical to account for the activity of all the components of NETs. This makes determining a given NET “dose” challenging; thus, many researchers use DNA content to define the “NET dose”. We found that NET NE and NET IL-1RA correlated with DNA content. The DNA concentration best-predicted disruption of the barrier function of HBE, providing further evidence for using DNA to determine NET concentrations. An alternative approach is to pool the NETs from multiple donors and average the protein activity differences.

The limitations of our study include the inability to control all the factors that influence an individual’s neutrophil function, including subclinical infections, which could explain some of the variation seen. Despite the fact that we chose a healthy population with no major medical problems, we could not limit all prescribed medications, nor could we control for stress or other inflammatory stimuli. Another limitation is the small sample size. For example, our study did not find significant heterogeneity in total NET DNA concentrations; however, given the variance estimates, we would expect to have an 80% detection power of significant heterogeneity in this analyte if we have 12 subjects each with eight repeated measurements. In this regard, our study generates important preliminary data to inform future studies related to NET biology and its impact on the epithelium. We acknowledge that the baseline phenotype of the donor neutrophil (including cellular maturity, activation, degranulation potential, etc.) could significantly alter the composition and concentration of NETs generated. Our future studies will focus on uncovering the degree to which these factors regulate observed differences in NETs.

## 4. Materials and Methods

### 4.1. Isolation of Human Neutrophils and NETs

The study protocol, No. 2016-3837, was approved by the University of Cincinnati institutional review board (IRB). Human peripheral blood was collected from healthy adult donors in sodium citrate vacuum tubes (Fisher Scientific, Waltham, MA, USA). Neutrophils were isolated by negative magnetic bead selection using MACSxpress Neutrophil Isolation Kit, Human (Miltenyi Biotec, Bergisch Gladbach, Germany). Negative bead selection was used to obtain highly enriched populations of neutrophils with a mean cell viability of 94.1%. In our hands, negative bead selection, though more costly, resulted in a homogenous cell population with less spontaneous activation of neutrophils compared to density gradient separation (Supplemental Figure S1A,B). Cell counts and viability were assessed using Trypan blue, 0.4% (Fisher Scientific). PMNs were suspended in RPMI-1640 media (GIBCO, Grand Island, NY, USA) with 3% fetal bovine serum (Fisher Scientific) at a concentration of 5 × 10^6^ cells/mL. Immediately after isolation, neutrophils were incubated at 37 °C, 5% CO_2_, 4 h in 100 mm tissue culture-treated Petri dishes (Fisher Scientific) with 25 nM PMA (Sigma-Aldrich, Burlington, MA, USA) added to stimulate NETosis [14,47]. Following incubation, the viscous NET layer was washed twice with PBS, scraped from the dish, and mixed vigorously. The sample was then centrifuged at 450× *g*, 10 m, 22 °C to remove cell debris. Cell-free NETs suspended in PBS were added to the apical surface of HBE.

### 4.2. Primary Epithelial Cell Culture

Primary HBEs were provided by the CCHMC CF Research Development Program Translational and Model Systems Cores. Cells were cultured as previously described [48]. Primary HBE from normal donors were obtained from University of North Carolina Airway Cell Core and were utilized at passages 2 to 3. HBE were differentiated and grown at ALI on 6.5 mm Transwell inserts (Costar, Corning, NY, USA). The apical surfaces of HBE were exposed to PBS or NETs suspended in PBS at 37 °C, 5% CO_2_ for 18 h, unless otherwise noted. Supernatants and cell protein lysate were stored for future testing. The use of primary HBE was approved by the CCHMC Pulmonary Biorepository Core and University of Cincinnati IRB.

### 4.3. Transepithelial Electrical Resistance 

TEER was measured across each epithelial cell layer in duplicate before and after exposure to test conditions and PBS control using an Epithelial Volt/Ohm meter EVOM2 and chopsticks electrode set STX3 (World Precision Instruments, Sarasota, FL, USA). Results are expressed as percent change from baseline TEER values [13].

### 4.4. Characterization of NETs and Cell Supernatants

Samples of NET isolates and HBE supernatants were collected, and aliquots stored at −80 °C. HBE were processed for protein lysates and stored at −20 °C (Complete Lysis-M Roche diagnostics). NET dosage was an average of extracellular dsDNA concentration (QuantiFluor ONE dsDNA System, Promega, Fitchburg, WI, USA) and total DNA content (SYTOX green nucleic acid stain), Fisher Scientific [49]. Protein content of NETs and cell lysates was determined using DC Protein assay (Bio-Rad, Hercules, CA, USA).

NETs were assayed for: LDH (Promega CytoTox 96 Cytotoxicity Assay); MPO activity (Cayman Chemical, Ann Arbor, MI, USA); NE, PR3, and CG activities (fluorescent resonance energy transfer (FRET) substrates Abz-APEEI MRRQ-EDDnp (NE), Abz-VADnV RDRQ-EDDnp (PR3), Abz-EPF WEDQ-EDDnp (CG); Peptide Institute, Osaka, JPN) [50]. Cytokine analysis was performed by Cincinnati Children’s Hospital Research Flow Cytometry Core per kit instructions (Milliplex MAP human protein panel, Millipore-Sigma).

### 4.5. Microscopy

Neutrophil and NET images were captured on a Zeiss Axioplan 2 or a Nikon A1R GaAsP inverted SP microscope using antibodies to NE (481001, Millipore Sigma, Burlington, MA, USA) and MPO (ab25989, Abcam, Cambridge, UK) with AF568 and AF488 secondary antibodies (Invitrogen, Carlsbad, CA, USA) and Hoechst nucleic acid stain (Invitrogen).

### 4.6. Statistical Analysis

Statistical analysis was performed using GraphPad Prism software v7.04 and SAS version 9.4 (SAS Institute, Inc., Cary, NC, USA). Subject means were compared using ordinary one-way ANOVA with Bonferroni corrections for multiple comparisons [51]. We assessed model assumptions using residual plots in the ANOVA models and used Kruskal–Wallis tests for outcomes that did not meet the normality assumption [52]. Variability between subjects’ values was measured by comparing standard deviations using the Brown–Forsythe test. Correlations were analyzed using Pearson correlations with best-fit lines by simple linear regression. We assessed the effects of age and gender in our model by using linear mixed-effects models that included age and sex as fixed effects while accounting for the repeated measures from the same subjects using subject-specific random effects. *p*-values ≥ 0.05 were not considered significant, while significant *p*-values are denoted by: <0.05 *, ≤0.01 **, ≤0.001 ***, ≤0.0001 ****. Graphs include data from at least three separate experiments, unless otherwise indicated. Each donor is represented by the same symbol across all graphs. 

### 4.7. Research Compounds

AAT (UniProt# P01009, Cayman Chemical), NEI (CAS#1448314-31-5, Cayman Chemical), SIV (CAS# 201677-61-4, Cayman Chemical), CGI (CAS# 429676-93-7, Cayman Chemical), PMA (CAS #16561-29-8, Millipore-Sigma).

### 4.8. Study Population

Healthy male and female adult subjects were enrolled in our study from 2020 to 2023 who met the following criteria: healthy, nonsmoker, no history of malignancy, no history of autoimmune disorders, no use of immunosuppressive agents (including steroids) within 90 days, no acute illness or antimicrobials within 2 weeks. Subjects had to abstain from alcohol or NSAIDs for 72 h prior to donation. A subject had consecutive blood draws at least one month apart. 

## 5. Conclusions

In conclusion, we demonstrate that NETs from multiple healthy donors have differing susceptibilities to protease inhibitors. These findings are clinically relevant, as anti-proteases (such as AAT) are commonly used therapies to treat lung diseases including alpha-1 antitrypsin deficiency. Future studies will elucidate if variable responses to these therapies could be due to the heterogeneity of NET content across human subjects that we report in this study. 

## Figures and Tables

**Table 1:** Study population characteristics. Peripheral blood was collected (n = 50, blood draws) from 12 healthy donors in 3–7 collections per subject over a 3.5-year study period from 2020–2023.

Subject	n	Gender	Age
**1**	6	F	49
**3**	4	F	43
**5**	4	M	52
**6**	7	F	63
**8**	4	M	34
**9**	3	M	24
**11**	7	M	21
**12**	3	M	64
**13**	3	M	52
**14**	3	F	24
**15**	3	F	25
**16**	3	F	23

## Data Availability

The data presented in this study are available on request from the corresponding author.

## Supplementary Materials

The following supporting information can be downloaded at: https://www.mdpi.com/article/10.3390/ijms25010525/s1.
