# Supplementary material for: Heterogeneity in Neutrophil Extracellular Traps from Healthy Human Subjects
Source: Int J Mol Sci. 2023 Dec 30;25(1):525. doi: 10.3390/ijms25010525 (PMC10779146; doi:10.3390/ijms25010525)
Supplement: Supplementary file 1 [file ijms-25-00525-s001.zip › ijms-2668886-supplementary.pdf]

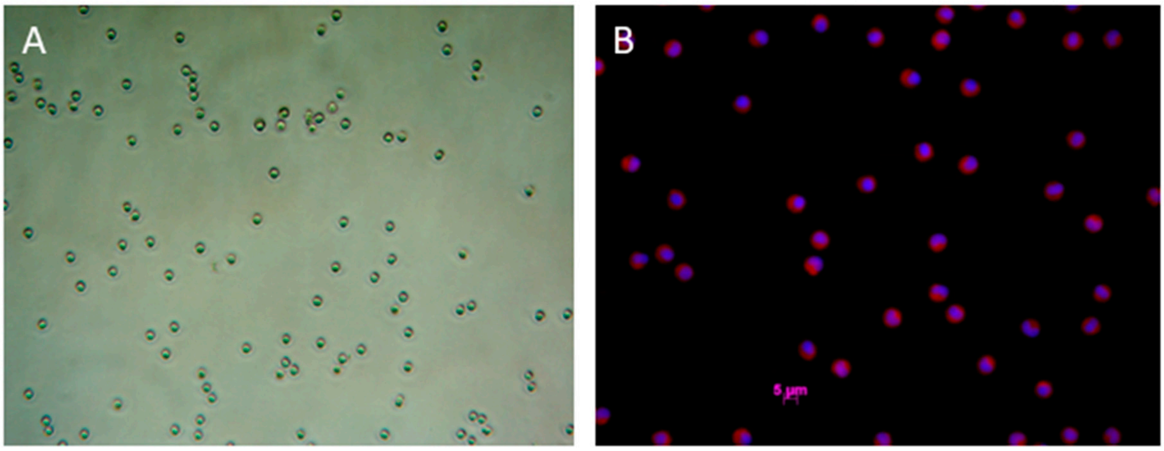

Figure S1. Purified neutrophils prior to stimulation to NETosis. (**A**) Bright field image of enriched unstained neutrophils obtained from donor blood using negative magnetic bead selection and suspended in PBS with 0.04% trypan blue. (**B**) Merged immunofluorescent image of unstimulated neutrophils at a concentration of 5 × 10^6/mL and a density of 1 × 10^5/cm^2 stained with NE antibody (AF568, red), and Hoechst (blue).

Table S1. Timing of subject blood donation.

Subject	Jan-Mar 2020	Apr-Jun	Jul-Sep	Oct-Dec	Jan-Mar 2021	Apr-Jun	Jul-Sep	Oct-Dec	Jan-Mar 2022	Apr-Jun	Jul-Sep	Oct-Dec	Jan-Mar 2023	Apr-Jun	Jul-Sep	Oct-Dec
1							◊	◊	◊	◊	◊			◊		
3	◊	◊				◊		◊								
5							◊	◊	◊		◊					
6							◊	◊	◊	◊	◊	◊		◊		
8	◊		◊		◊	◊										
9							◊	◊				◊	◊			
11							◊		◊		◊	◊		◊	◊	◊ ◊
12															◊	◊ ◊
13															◊	◊ ◊
14															◊	◊ ◊
15															◊	◊ ◊
16															◊	◊ ◊
